# Choosing Appropriate Candidates for Left Atrial Appendage Occlusion

**DOI:** 10.31083/j.rcm2412360

**Published:** 2023-12-25

**Authors:** Chengxiang Zhang, Hao Lu, Yuansong Zhu

**Affiliations:** ^1^Department of General Medicine, The First Affiliated Hospital of Chongqing Medical University, 400016 Chongqing, China; ^2^Department of Cardiology, The First Affiliated Hospital of Chongqing Medical University, 400016 Chongqing, China

**Keywords:** atrial fibrillation, AF, left atrial appendage, LAA, left atrial appendage occlusion, left atrial appendage closure, stroke

## Abstract

Atrial fibrillation (AF) is one the most prevalent arrhythmias globally and is associated with a significantly higher risk of morbidity and mortality, including an up to five-fold increase in risk of stroke. While oral anticoagulation therapy remains the standard approach for stroke prevention in nonvalvular AF, left atrial appendage occlusion (LAAO) has emerged as a viable alternative for patients who are intolerant to long-term oral anticoagulation therapy. However, selecting appropriate candidates for LAAO requires a comprehensive evaluation that considers various clinical factors, although this presents a challenge in clinical practice. This review aims to provide an overview of the current recommendations for patient selection in LAAO procedures and the key factors that need to be considered both before and after the procedure, as well as the ongoing advancements in this field that may facilitate the selection of patients for LAAO.

## 1. Introduction

Atrial fibrillation (AF) is the most common clinically significant cardiac arrhythmia [1, 2]. AF is associated with a five-fold increase in risk for stroke, contributing to approximately 15% of all strokes [3]. Oral anticoagulation (OAC) therapy is the standard strategy for preventing thromboembolic events in AF patients. However, long-term OAC cannot be achieved in a significant proportion group of patients due to an increased risk of bleeding or patient non-compliance. A large-scale study using the American National Cardiovascular Data Registry revealed that only 44.9% of patients with AF received OAC therapy [4]. European data further demonstrated that only 50% of AF patients are receiving OAC therapy, with the discontinuation rate reaching as high as 70% after a 5-year follow-up [5].

Previous studies have established that the left atrial appendage (LAA) is responsible for approximately 90% of thromboembolic events in patients with AF, providing a theoretical foundation for the development of the left atrial appendage occlusion (LAAO) procedure [6]. Subsequently, a significant increase in the number of studies aiming to investigate the efficacy and safety of the LAAO has occurred over the past decade. However, to ensure the benefits of the procedure in preventing thromboembolic events outweigh the potential risks of periprocedural and postprocedural adverse events, the process of selecting appropriate candidates necessitates further extensive research and discussion. Therefore, the objective of this review is to provide an overview of the key factors that need to be considered both prior to and following LAAO, thereby providing guidance for its use in clinical practice.

## 2. Current Evidence on Left Atrial Appendage Occlusion

Situated at the left border of the left ventricle and pulmonary outflow tract, the LAA is a remnant accessory structure in the primitive left atrium during embryonic development. The LAA plays a role in regulating left atrial pressure and has potential hemodynamic implications [7]. Aside from its physiological functions, the LAA is also considered a high-risk area for the formation of blood clots due to the relative stasis owing to its shape and trabeculations [8]. LAAO is a novel transcatheter technique that emerged at the beginning of this century and has gained rapid development in recent years. This technique involves delivering a closure device to the LAA via a catheter, covering or sealing off the LAA from the circulation, to prevent LAA-related thromboembolic events without increasing the risk of bleeding related to long-term OAC therapy.

The concept of LAAO first appeared in 1949 as a surgical procedure, while the first percutaneous LAAO in human patients was performed in 2001 [9]. In 2002, Sievert *et al*. [10] performed LAAO on 15 chronic AF patients at high risk of stroke. With the exception of one patient who experienced pericardial bleeding during the procedure, the device was successfully implanted in all the other patients. A follow-up after one month showed the device was positioned stably without dislodgement, perforation, or device-related embolization. This study provided initial evidence for the feasibility of percutaneous LAAO procedures. The efficacy and safety of LAAO in comparison to warfarin, in patients who are eligible for both strategies, have been subsequently investigated in two randomized clinical trials: the PROTECT-AF (Watchman left atrial appendage system for embolic protection in patients with atrial fibrillation) trial and the PREVAIL (Watchman LAA closure device in patients with atrial fibrillation versus long term warfarin therapy) trial [11, 12]. The long-term data from the PROTECT-AF trial demonstrated the noninferiority of the LAAO to warfarin in preventing the combined outcomes of stroke, systemic embolism (SE), and cardiovascular death, as well as a superiority for cardiovascular and all-cause mortality [11]. Although the PREVAIL study failed to show noninferiority of LAAO regarding the first composite co-primary endpoint of stroke, SE, or cardiovascular/unexplained death, the second co-primary endpoint of post-procedure stroke/SE did achieve noninferiority [12]. Accordingly, together with real-world data following these two trials, LAAO has become an effective alternate strategy for stroke prophylaxis in patients with nonvalvular AF.

The LAAO procedure is associated with complications, including device embolization, pericardial effusion, cardiac perforation, major bleeding, and vascular complications. However, a learning curve has been found as a result of accumulated procedural volumes and along with improved device technology, as reflected by the reduced complication rate over time [13, 14, 15]. Furthermore, future research focusing on modifying the process of patient selection may further enhance the safety of the procedure.

## 3. Indications for LAAO Outlined by Current Clinical Guidelines

The recommendations for LAAO in the current guidelines are as follows: The 2020 European Society of Cardiology guideline for AF management stated that LAAO may be considered for stroke prevention in patients with AF and contraindications for long-term OAC treatment, such as intracranial bleeding without a reversible cause, and was listed as a class IIb recommendation [16]. Furthermore, the American College of Cardiology/American Heart Association guideline was updated in 2019 and indicated that OAC remains the preferred therapy for stroke prevention while recognizing that LAAO provides an alternative for patients who are not suitable candidates for long-term OAC, as a result of the propensity for bleeding or poor drug tolerance/adherence [17]. This recommendation is also classified as class IIb.

As reflected by the recommendations in the current guidelines, the driving force for considering LAAO remains the benefits of stroke prevention against the possible adverse outcomes related to OAC. However, the specific criteria for patient selection have remained vague, owing to limited randomized controlled trial data. Clinical factors in favor of or against LAAO under certain clinical circumstances will be discussed in detail below.

## 4. Preprocedural Considerations

### 4.1 Age

Age is a crucial factor that should be considered before undergoing the LAAO procedure. As age is included in the ${\mathrm{CHA}}_{2}$${\mathrm{DS}}_{2}$-VASc and HAS-BLED scores, older aged patients are at high risk of both thromboembolic events and bleeding, which theoretically makes them candidates for LAAO. However, older patients with many comorbidities have competing causes of mortality, and therefore, may not profit from the procedure to the same extent as younger patients with fewer comorbidities due to the limited expected longevity. Observational studies have shown that while LAAO can reduce the risk of ischemic stroke in all age groups, the 2-year mortality rate increases significantly with age [18]. Real-world registries have reported higher mortality rates compared to randomized controlled trials [19, 20, 21]. After conducting multivariable analysis, a study showed a 15.5% mortality rate in consecutive patients undergoing LAAO over a 10-year period, while older age was identified as an important predictor of early death [21]. Another French registry documented a 1-year mortality rate of 7.4%, of which 82% was non-cardiac-related [19]. These data suggested that LAAO is being used in older and sicker patients in real-life treatments compared to clinical trials and that early death is not uncommon. Furthermore, the postprocedural antithrombotic regimen should also be carefully tailored as older patients have a higher tendency for bleeding despite receiving the same antithrombotic regimen [22]. In theory, LAAO is a procedure from which patients may derive more benefits over time, considering the expected longevity and cumulative protective effect from stroke. Thus, additional research is needed to thoroughly examine the influence of aging on LAAO outcomes and provide personalized recommendations that consider the age and expected longevity of patients.

### 4.2 Gender

Considerable gender differences, in terms of epidemiology, risk factors, treatment, and prognosis, exist in AF patients. While men have a higher cumulative risk of developing AF, women appear to be at a higher risk of AF-related stroke, which has not been modified by anticoagulation therapy [23, 24, 25]. Additionally, various reasons depict that female patients are less likely to receive OAC, including the underestimation of the thromboembolic risk by clinicians, the shared decision-making support and risk framing experienced by women, and nonmedical reasons, such as time and cost [26, 27]. The higher thromboembolic risk and undertreatment support illustrate that women with AF are suitable surrogates for LAAO. Although there is only a limited number of studies targeting whether gender has any effect on the long-term outcomes after LAAO, the current data appear to suggest that LAAO could introduce a neutralizing effect and provide similar efficacy for both sexes in stroke prevention—this comes after several previous studies found no significant differences in the 1–2-year stroke and mortality rates between sexes [28, 29, 30, 31]. However, special attention should be given to women during the periprocedural phase as they may experience more procedural adverse events, including pericardial effusion, major bleeding, and vascular complications [28, 29, 30, 32]. This could be attributed to factors such as smaller and thinner atrial appendage walls, more friable tissue, smaller vessel diameter, and other unmeasured confounding factors. These results emphasize the need for mitigating strategies to optimize the short-term safety of LAAO in women.

### 4.3 Anatomical Considerations

#### 4.3.1 LAA Thrombus

It has been reported that in patients scheduled for AF ablation, who have a ${\mathrm{CHADS}}_{2}$ score of 4–6, as assessed by transesophageal echocardiography, the incidence of LAA thrombus can be as high as 11%, despite adequate anticoagulation [33]. Consequently, the prevalence of LAA thrombus may be even higher in patients referred for LAAO, as they typically have a high ${\mathrm{CHA}}_{2}$${\mathrm{DS}}_{2}$-VASc score and often cannot be effectively treated by OAC. The presence of a thrombus in the LAA has been considered a contraindication for percutaneous LAAO, as the manipulation of sheaths, guidewires, or devices in the LAA may lead to systemic embolization [34]. Therefore, patients with LAA thrombus have been excluded from large-scale LAAO trials. As the experience of the operators has improved over time, recent publications have emerged reporting attempts to perform LAAO procedures in patients with thrombus in the LAA [35, 36, 37]. Despite the initial safety and efficacy being achieved, these data were based on a limited sample size with limited follow-up. Moreover, the procedure was performed by experienced operators with an appropriate cerebral protection system, which was adopted by and restricted to highly selected patients with recurrent LAA thrombus, despite sufficient anticoagulation therapy. Another echocardiographic phenomenon that poses an increased risk for thromboembolic events is spontaneous echo contrast (SEC) [38]. However, only a few recent studies have investigated the impact of SEC on outcomes following LAAO. These studies have shown that the presence of SEC does not appear to be associated with an increased risk of thromboembolic events during the follow-up, although it does slightly raise the incidence of device-related thrombus [39, 40]. Nevertheless, more data are necessary before any indications of LAAO are extended to patients with LAA thrombus or SEC.

#### 4.3.2 LAA Gross Morphology

**Fig. 1.**
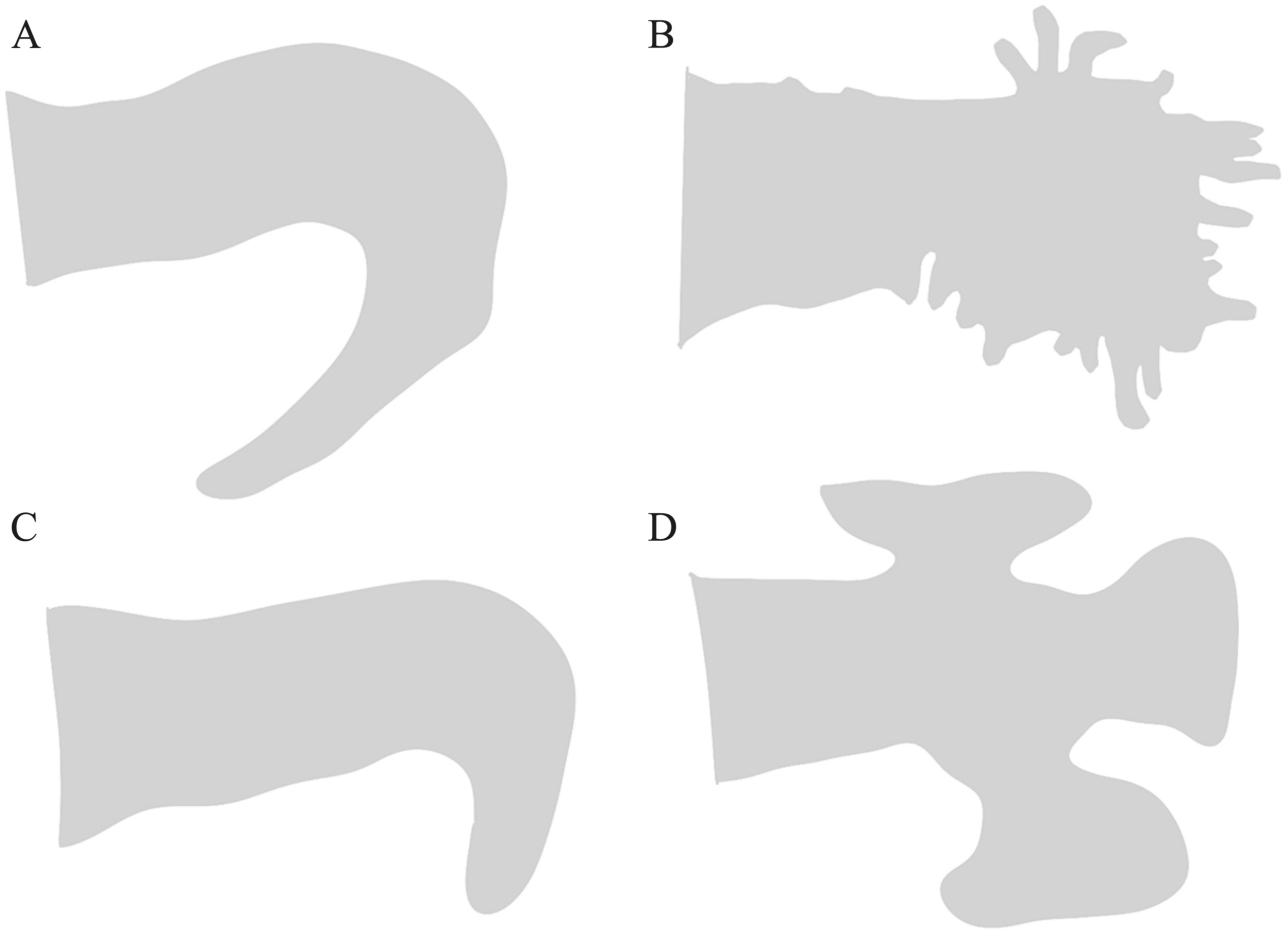
**The gross morphologies of the LAA**. (A) Chicken wing. (B) Cauliflower. (C) Windsock. (D) Cactus. LAA, left atrial appendage.

The distribution of different LAA anatomies is heterogeneous in the existing literature. However, the gross LAA morphologies in AF patients could be classified into chicken wing, cactus, windsock, and cauliflower (Fig. 1) [41, 42]. Fastner *et al*. [42] analyzed the LAA morphologies in 562 patients undergoing LAAO from the German LAARGE registry and added a group of atypical morphologies in addition to the four typical morphologies. It was demonstrated that procedural success as well as the complication rates of LAAO remained similar among the typical morphologies (≥97.5%), while a lower implantation success rate was only seen in atypical morphologies (94%). Although it is crucial to obtain preprocedural accurate sizing and knowledge of the gross type of the LAA to improve the procedural success rate and reduce the frequency of complications, there is no reason to preclude patients from LAAO solely based on the current data on the gross morphology of the LAA.

#### 4.3.3 LAA Orifice and Depth

Preprocedural assessment of the LAA orifice and depth is crucial to ensure optimal size selection and the safe placement of the device. Previous autopsy and imaging studies revealed that the LAA ostium is usually round or in an elliptical-shaped variant [43, 44]. Different LAAO devices may have varying size requirements and they are designed to fit and seal the LAA effectively. The main differences between the two current major types of occlusive devices lie in their shape and method of placement. The Watchman occluder is positioned 10 mm from the ostium and does, therefore, not completely cover the LAA. The Amplatzer Cardiac Plug (ACP) device consists of a distal anchoring lobe that is placed about 10–15 mm away from the LAA ostium and a proximal disc that covers the LAA entrance. The recommended maximal diameter of the LAA orifice is between 17 and 31.9 mm for the Watchman device and 12.6–28.5 mm for the ACP device [45]. When using the ACP system, it is crucial to carefully determine the appropriate size of the landing zone for the anchoring lobe, as the device should not be adopted when this area is less than 10 mm in width [46]. For the Watchman device, it is essential that the length of the LAA exceeds the maximum ostial diameter to guarantee a safe landing zone.

#### 4.3.4 LAA Pectinate Muscles

The endocardial surface of the LAA is lined with a complex network of fine pectinate muscles. Autopsy studies have shown that these muscles are usually thicker than 1 mm in 95–97% of hearts, particularly in individuals in the first or last decade of life [47]. Some specimens demonstrate additional muscular trabeculations that extend downwards from the LAA to the vestibule of the mitral valve. These extra myocardial bands are formed by a small set of posterior pectinate muscles starting from the myocardial bundles, thereby embracing the LAA. In hearts with these additional muscles, the areas between the trabeculae and the atrial walls become extremely thin [45, 48]. It is important to note that these anatomical features can pose challenges during LAA interventions. For instance, when performing maneuvers such as inserting catheters or delivery sheaths, they can become lodged in the pits and troughs formed by these muscles, and increase the risk of cardiac perforation. Furthermore, the presence of large muscular trabeculations near the LAA ostium may contribute to leaks around the device after implantation, which can compromise the effectiveness of the procedure and require further management.

#### 4.3.5 Interatrial Septum

During the implantation of the LAAO devices, the structure of the interatrial septum (IAS) should be considered, as they are accessed via the transseptal pathway. The IAS has a variable oblique course, and its angle to the sagittal plane depends on the size of the atrial chambers and the orientation of the heart [45, 47]. Therefore, fluoroscopic angulations for transseptal punctures must be individualized. An inferior–posterior transseptal puncture of the fossa ovalis is preferred to enable a frontal approach to the LAA ostium and avoid accidental puncture of surrounding structures, which could lead to hemopericardium [45]. Precisely evaluating the size and morphology of the oval fossa and any associated patent foramen ovale (PFO) is crucial during the procedure. In cases of PFO, where the anterosuperior aspect of the rim is not sealed, it would be more appropriate to perform a lower transseptal puncture. However, other conditions, such as the presence of interatrial communications with patches or occluder devices, IAS aneurysm, or thickened fibrotic septum after prior transseptal interventions, can present challenges in achieving trans-atrial access to the left atrium [45, 49]. In such cases, detailed anatomical evaluation using transesophageal echocardiography or intracardiac echocardiography can aid in planning the transseptal approach.

### 4.4 Comorbidities

Many patients referred for LAAO have significant comorbid conditions, making it crucial to evaluate the benefits versus risks of the procedure while engaging in any medical decision-making. A recent study, which used the US National Inpatient Sample database, revealed that patients undergoing LAAO could have an average of 12.3 comorbidities [50]. The comorbidity burden was measured using validated global measurements, such as the Charlson comorbidity index (CCI), Elixhauser comorbidity score (ECS), and the ${\mathrm{CHA}}_{2}$${\mathrm{DS}}_{2}$-VASc score. Patients whose CCI, ECS, and ${\mathrm{CHA}}_{2}$${\mathrm{DS}}_{2}$-VASc were higher had a significantly increased risk of in-hospital major adverse events [50]. These findings highlight the necessity of assessing the benefits and risks in patients with a heavy burden of comorbidities. However, there are additional aspects that require consideration, as detailed in the following sections.

#### 4.4.1 Rheumatic Heart Disease

Patients with rheumatic heart disease, specifically those with AF that is related to mitral stenosis or mechanical heart valves, are not suitable candidates for LAAO. These patients exhibit potentially different mechanisms of thrombus formation compared to patients with nonvalvular AF, yet OAC remains the cornerstone therapy [51]. A systemic review by Blackshear *et al*. [52] revealed that only 57% of patients with rheumatic AF and documented left atrial thrombus had left atrial thrombus located in the LAA compared with 90.5% in nonvalvular AF.

#### 4.4.2 Active Bleeding

Patients with active bleeding or those still in the hemorrhagic transformation stage after an acute ischemic stroke should have LAAO postponed owing to the periprocedural antithrombotic drugs that are required for the procedure. It is important to note that the optimal time of LAAO that should be individualized in these patients, and that, such conditions do not constitute contraindications for LAAO. In fact, patients who underwent LAAO with previous intracranial bleeding or stroke on adequate OAC presented similar safety outcomes compared to patients without the same history [53]. In patients with previous major gastrointestinal bleeding, although LAAO was associated with higher procedural major bleeding events, the implantation of LAAO significantly reduced the annual stroke or transient ischemic attack events [54].

#### 4.4.3 Alternate Diseases Requiring OAC

Patients with AF and certain alternate diagnoses that require indefinite anticoagulation should be excluded from consideration for LAAO. These conditions include a hypercoagulable state (factor V Leiden, prothrombin 20210 gene mutation, protein C or S deficiency, or antiphospholipid syndrome), history of heparin-induced thrombocytopenia, deep venous thrombosis, or pulmonary emboli [55]. Nevertheless, LAAO may serve as an option in patients with “resistant stroke”, i.e., patients who still experience thromboembolic events with a high likelihood of the embolism originating from the LAA, despite adequate OAC, since it will avoid the necessity for chronic dual or triple medical therapy [56].

## 5. Postprocedural Considerations

Although the efficacy and safety of LAAO have been demonstrated in randomized controlled trials and multiple registries with medium- and long-term data, there is still a significant incidence of device-related thrombosis (DRT; 3.7%–7.2%), and the increased risk of these thromboembolic events need to be emphasized [56]. Several studies have investigated the potential risk factors associated with DRT and found the results to be multifactorial [57, 58]. It remains uncertain whether DRT is primarily influenced by patient factors, procedural factors (some of which have been discussed earlier in this review), or the type and duration of the postprocedural antithrombotic regimen. Currently, the antithrombotic management after LAAO has never been evaluated in a randomized trial, and recommendations are made based on historical studies. While a thorough comparison of different antithrombotic regimens is beyond the scope of this review, it is important to at least note that a strategy with no antithrombotic therapy at all is not appropriate for patients undergoing LAAO. Studies have shown that both OAC and antiplatelet therapy were independently associated with a reduced risk of DRT [59]. Therefore, individualized assessment of the benefits and risks of DRT and postprocedural antithrombotic therapy should guide the selection of candidates for LAAO treatment.

## 6. Prospects for the Future of LAAO

Future research endeavors have the potential to provide valuable insights into the selection of appropriate candidates for LAAO procedures by exploring the following aspects. First, several recently published trials have demonstrated the noninferior efficacy and superior safety of LAAO compared to NOAC (non-vitamin K antagonist oral anticoagulant), with more data in this area expected to further strengthen the role of LAAO in the management of AF, in the era of NOAC [60, 61, 62]. Furthermore, the introduction of next-generation LAAO devices, such as the Watchman-FLX by Boston Scientific, offers promising advancements [63]. These devices feature an improved seal, closed distal cell design, and reduced metal exposure; enhancements that may potentially reduce the anatomical requirements of LAA and lower the risk of periprocedural cardiac effusions and postprocedural DRT. Consequently, these advancements have the potential to expand the applicability of LAAO to a broader range of patients in the future.

## 7. Conclusions

LAAO has emerged as an important approach to address the unmet need for thromboembolic event prevention in the management of AF patients. Careful planning is essential for an LAAO procedure. The selection of patients for LAAO should be approached by considering both the advantages of preventing thromboembolic events and the potential risks associated with the procedure and its aftermath. The continuous development of advanced LAAO devices, along with the growing body of evidence demonstrating the efficacy and safety of LAAO, in comparison to conventional approaches, is anticipated to enhance the progress and optimization of AF treatment.

## Abbreviations

ACP, Amplatzer cardiac plug; AF, atrial fibrillation; CCI, Charlson comorbidity index; DRT, device-related thrombus; ECS, Elixhauser comorbidity score; IAS, interatrial septal; LAA, left atrial appendage; LAAO, left atrial appendage occlusion; OAC, oral anticoagulation; PFO, patent foramen ovale; PREVAIL, Watchman LAA closure device in patients with atrial fibrillation versus long term warfarin therapy; PROTECT-AF, Watchman left atrial appendage system for embolic protection in patients with atrial fibrillation; SE, systemic embolism; SEC, spontaneous echo contrast.
